# Effects of obesity on DNA methylation and DNA methyltransferases in the reproductive system

**DOI:** 10.1007/s00418-026-02481-x

**Published:** 2026-04-26

**Authors:** Gozde Sukur, Ozgur Cinar, Fatma Uysal Cinar

**Affiliations:** 1 Department of Molecular Biology and Genetics, Faculty of Science, Gebze Technical University, 41400 Gebze, Kocaeli Turkey; 2 Department of Histology and Embryology, Ankara University School of Medicine, 06080 Ankara, Altindag Turkey; 3 Department of Histology and Embryology, Ankara Medipol University School of Medicine, 06080 Ankara, Altindag Turkey

**Keywords:** DNA methylation, DNA methyltransferases, Obesity, Infertility, Epigenetics

## Abstract

Obesity is increasingly recognized as a condition with profound systemic and epigenetic consequences. However, the molecular mechanisms linking obesity to reproductive dysfunction remain incompletely understood. Accumulating evidence suggests that epigenetic regulation, particularly DNA methylation, contributes to and is modified by obesity, thereby influencing gene expression in gonadal tissues and germ cells. DNA methylation is essential for normal oogenesis and spermatogenesis and is mediated by DNA methyltransferases (DNMTs), whose expression and activity are tightly regulated throughout germ cell development. This review synthesizes current experimental and clinical evidence regarding obesity-associated alterations in DNMT expression and DNA methylation patterns across female and male reproductive systems and examines how these epigenetic disruptions contribute to infertility. Obesity is most frequently associated with a hypermethylated epigenetic landscape in female reproductive tissues, whereas the male germline more commonly exhibits global or locus-specific hypomethylation, reflecting sex-specific epigenetic responses to metabolic stress. Studies spanning ovarian tissue, oocytes, sperm, and early embryos demonstrate that obesity-induced DNMT dysregulation and DNA methylation remodeling disrupt transcriptional programs governing folliculogenesis, spermatogenesis, and embryo development.

## Introduction

Obesity, as defined by the World Health Organization (WHO), is characterized by the excessive or abnormal accumulation of body fat that poses a risk to health. Recognized as a complex and multifactorial disease, obesity is strongly associated with adverse systemic consequences and constitutes a major risk factor for noncommunicable disorders, including type 2 diabetes, cardiovascular disease, hypertension, respiratory disorders, and several cancers (Alberti et al. 2009; Sultan et al. 2020). The etiology of obesity reflects a dynamic interplay among genetic predisposition, familial background, socioeconomic and sociocultural determinants, sedentary lifestyles, and chronic consumption of energy-dense diets (Hruby and Hu 2015; Ambele et al. 2020).

Within this framework, epigenetic mechanisms have emerged as critical mediators linking environmental exposures to gene expression regulation, thereby contributing to obesity development and its downstream metabolic complications (Singh et al. 2017; Huang et al. 2018). Accordingly, increasing attention has been directed toward understanding how epigenetic modifications shape obesity-associated pathophysiology (Tzika et al. 2018; van Dijk et al. 2015). Among these regulatory layers, DNA methylation has gained particular prominence owing to its central role in transcriptional control, genomic stability, and cellular differentiation.

Epigenetic regulation is equally indispensable for the precise orchestration of gene expression during mammalian oogenesis and spermatogenesis. DNA methylation, a major epigenetic mechanism, involves the covalent addition of a methyl group to cytosine residues and is catalyzed by DNA methyltransferases (DNMTs). In mammals, six DNMT family members have been identified: DNMT1, DNMT2, DNMT3A, DNMT3B, DNMT3C, and DNMT3L, each contributing distinct yet coordinated functions in the establishment and maintenance of DNA methylation patterns. DNMT1 primarily mediates maintenance methylation during DNA replication (Turek-Plewa and Jagodzinski 2005), DNMT3A and DNMT3B regulate de novo methylation, DNMT3C safeguards the male germline genome against transposon activation (Barau et al. 2016), DNMT3L functions as a regulatory cofactor essential for imprint establishment, and DNMT2 predominantly methylates transfer RNA (tRNA) (Goll et al. 2006). A detailed overview of DNMT functions and representative knockout phenotypes is presented in Table 1.

**Table 1:** Functions of mammalian DNA methyltransferases (DNMTs) and representative knockout phenotypes

DNMT	Primary function	Role in germ cells	Knockout phenotype/consequences	Key references
DNMT1	Maintenance methylation	Maintains methylation during DNA replication, imprint stability	Embryonic lethality, global hypomethylation, loss of imprinting	Li et al. (1992), Uysal et al. (2016)
DNMT2	RNA methyltransferase	RNA stability	Loss of RNA methylation, no major embryonic phenotype	Goll et al. (2006)
DNMT3A	De novo methylation	Establishment of new methylation marks during germ cell development, maternal and paternal imprinting	Embryonic lethality, imprinting defects, spermatogenic arrest, azoospermia	Okano et al. (1999), Uysal et al. (2016)
DNMT3B	De novo methylation	Establishment of new methylation marks during early development, methylation of repetitive elements	Embryonic lethality, Immunodeficiency, Centromeric instability and Facial anomalies (ICF) syndrome	Xu et al. (1999)
DNMT3C	Transposon suppression (testis-specific)	Protects the male germline genome from retrotransposon activation	Male infertility due to transposon derepression	Barau et al. (2016)
DNMT3L	Regulatory cofactor (noncatalytic)	Required for genomic imprint establishment and normal spermatogenesis	Abnormal imprinting, infertility, azoospermia	Bourc’his et al. (2001), Hata et al. (2002)

Despite accumulating evidence linking obesity to epigenetic remodeling, relatively few studies have directly examined how obesity modulates DNMT expression and global DNA methylation dynamics within gonadal tissues. Moreover, the molecular pathways connecting obesity-associated metabolic disturbances to reproductive dysfunction remain incompletely resolved (Crujeiras and Casanueva 2015). Given that obesity profoundly disrupts systemic metabolic, inflammatory, and endocrine homeostasis, it is plausible that these disturbances interfere with DNMT-mediated methylation processes governing germ cell development.

**Fig. 1:**
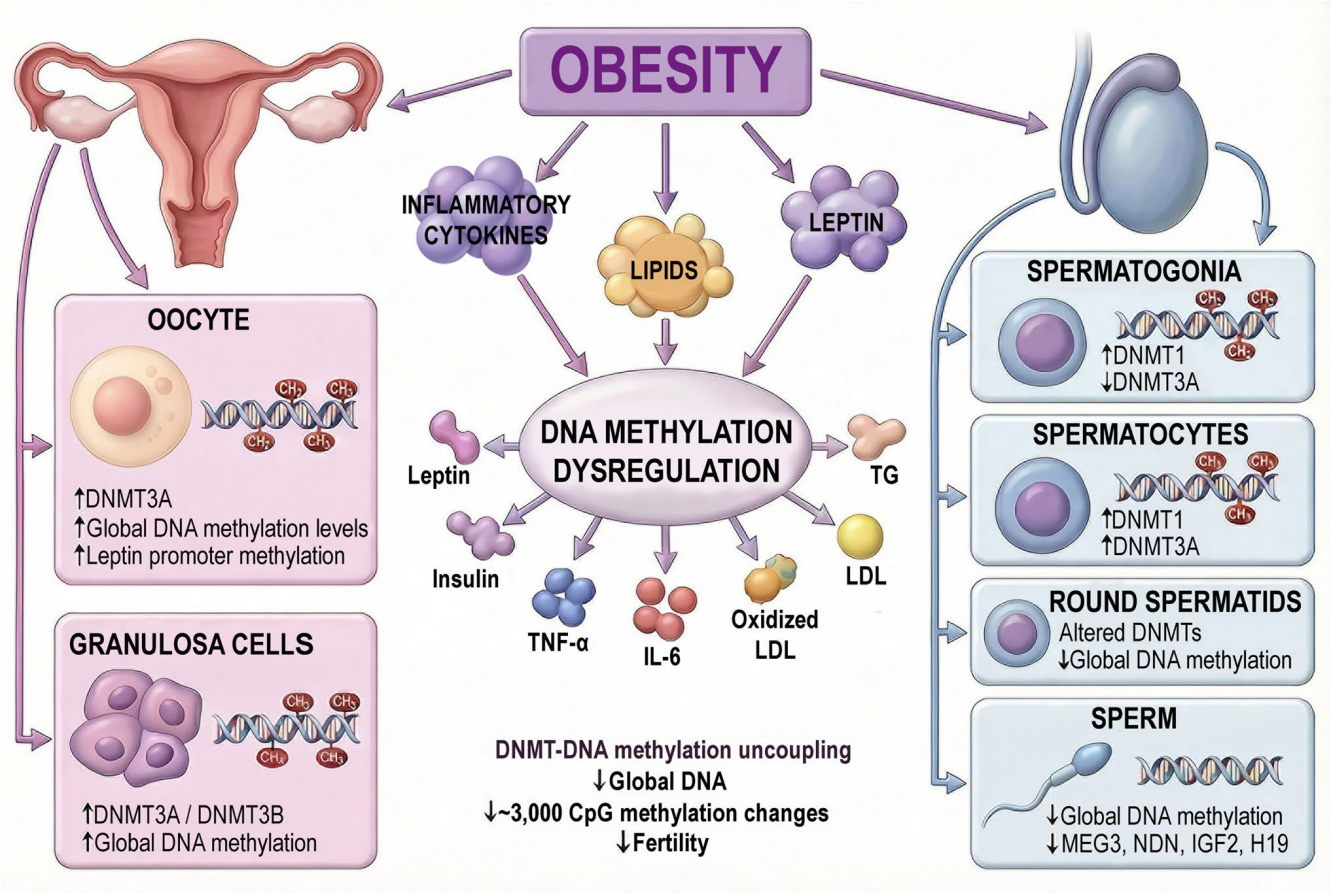
Obesity leads to hypermethylation in female and hypomethylation in male reproductive cells by altering DNMT expression. These changes disrupt normal epigenetic regulation and contribute to infertility. ↑, increase; ↓, decrease. *DNMT* DNA methyltransferase, *IL-6* interleukin-6, *LDL* low-density lipoprotein, *TG* triglyceride

Accordingly, this review synthesizes current experimental and clinical evidence describing obesity-associated alterations in DNMT expression and DNA methylation patterns across female and male reproductive systems. The overall relationship among obesity, DNMT dysregulation, and sex-specific DNA methylation outcomes is summarized in Fig. 1, which presents an integrated mechanistic framework linking metabolic imbalance to reproductive dysfunction. Furthermore, we integrate findings from reproductive tissues, gametes, and early embryos to delineate how DNMT dysregulation and DNA methylation remodeling mechanistically link metabolic status to fertility outcomes.

## The effect of obesity on the female reproductive system

Obesity is widely recognized as a major modifier of female reproductive physiology, exerting detrimental effects on both ovarian and endometrial function (Silvestris et al. 2018). Excess adiposity is associated with a spectrum of reproductive complications, including menstrual irregularities, anovulation, infertility, increased miscarriage risk, and reduced success rates in assisted reproductive technologies.

At the systemic level, women with obesity frequently exhibit insulin resistance, hyperleptinemia, and hyperandrogenism (Silvestris et al. 2018; Dag and Dilbaz 2015). These metabolic and endocrine disturbances collectively disrupt hypothalamic–pituitary–gonadal (HPG) axis homeostasis, resulting in altered gonadotropin secretion and steroid hormone imbalance (Silvestris et al. 2018). Such dysregulation is commonly accompanied by elevated circulating levels of luteinizing hormone (LH), androstenedione, estrone, insulin, triglycerides, and very-low-density lipoprotein (VLDL), together with reduced high-density lipoprotein (HDL) concentrations (Silvestris et al. 2018; Bellver et al. 2007). These hormonal and metabolic perturbations contribute directly to impaired folliculogenesis, ovulatory dysfunction, and compromised endometrial receptivity (Bellver et al. 2007; Silvestris et al. 2018).

Beyond endocrine disruption, accumulating evidence indicates that obesity-associated metabolic stress also reshapes the ovarian epigenetic landscape (Silvestris et al. 2018; Ge et al. 2014b). In particular, alterations in DNA methylation patterns and dysregulation of DNA methyltransferases (DNMTs) have emerged as potential molecular mediators linking obesity to defective follicular development, reduced oocyte competence, and impaired endometrial function (Ge et al. 2014b; Xu et al. 2016).

Importantly, interventional studies support the functional reversibility of these effects. Weight reduction has been associated with significant improvements in ovulatory function and fertility outcomes (Dag and Dilbaz 2015). Clark et al. demonstrated that even modest weight loss markedly improved ovulation rates, conception probability, and pregnancy outcomes in anovulatory women with infertility who have obesity (Clark et al. 1998).

Because ovarian tissue represents the primary microenvironment governing oocyte development, the following subsection focuses on obesity-associated alterations in DNMT expression and DNA methylation dynamics within ovarian and other female reproductive tissues.

### Effects of obesity on DNA methylation and DNA methylation enzymes in ovarian and reproductive tissue

The high prevalence of obesity among women of reproductive age is closely linked to impaired fertility (Hohos and Skaznik-Wikiel 2017). Studies examining the effect of obesity on folliculogenesis have reported inconsistent findings, including increased (Sohrabi et al. 2015; Wang et al. 2014), decreased (Balasubramanian et al. 2012; Brothers et al. 2010; Ma et al. 2016; Tortoriello et al. 2004; Wu et al. 2014), or unchanged follicle numbers (Lai et al. 2014; Skaznik-Wikiel et al. 2016). These discrepancies likely reflect differences in dietary fat composition, fat percentage, duration of exposure, and strain-specific metabolic responses.

Nevertheless, studies employing high-fat diets (HFDs) ranging from 45% to 60.9% fat, compared with control diets containing 4.8–18% fat, consistently demonstrate accelerated depletion of the early follicle pool (Skaznik-Wikiel et al. 2016; Sohrabi et al. 2015; Wang et al. 2014) and increased follicular abnormalities (Jungheim et al. 2010; Ma et al. 2016; Wang et al. 2014). Collectively, these findings suggest that prolonged exposure to high dietary fat content promotes premature ovarian aging and functional decline.

**Fig. 2:**
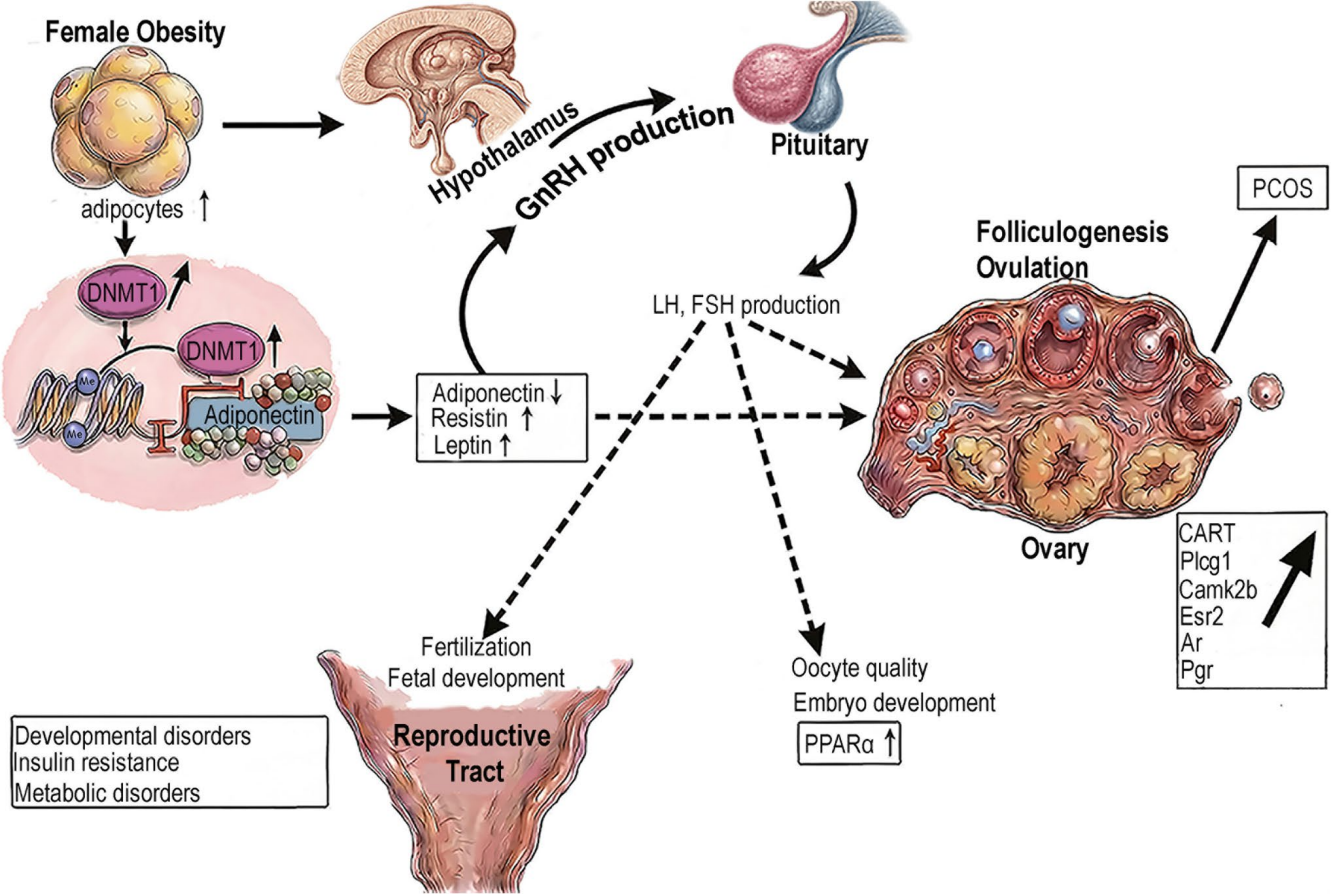
Obesity alters adipokine levels, disrupts the HPG axis, and impairs folliculogenesis and oocyte quality. Hypermethylation of PCOS-related genes has also been reported. Solid arrows indicate direct effects; dashed arrows indicate indirect effects. ↑, increase; ↓, decrease. *HPG* hypothalamic–pituitary–gonadal, *LH* luteinizing hormone, *FSH* follicle-stimulating hormone, *PCOS* polycystic ovary syndrome, *Plcg1* phospholipase C gamma 1, *Camk2b* calcium/calmodulin-dependent protein kinase II beta, *Esr2* estrogen receptor 2, *Ar* androgen receptor, *Pgr* progesterone receptor, *PPAR-α* peroxisome proliferator-activated receptor alpha

Importantly, obesity-associated metabolic disturbances are increasingly recognized to intersect with DNMT regulation and DNA methylation dynamics. These metabolic and epigenetic alterations converge on follicular activation, oocyte maturation, and imprint establishment, as schematically shown in Fig. 2.

In our previous studies, we investigated the effects of HFD-induced obesity on DNMT expression and global DNA methylation in mouse ovaries (Sukur et al. 2023; Uysal et al. 2023). We observed significant upregulation of DNMT1, DNMT3A, DNMT3B, and DNMT3L, accompanied by increased global DNA methylation across all stages of follicular development in HFD-exposed ovaries. These results suggest that obesity promotes a hypermethylated ovarian environment, which may disrupt transcriptional programs essential for normal follicular growth.

Beyond the ovary, altered DNA methylation and DNMT expression in the obese uterus may also contribute to implantation failure, indicating that obesity-induced epigenetic remodeling affects multiple female reproductive compartments (Bozdemir et al. 2024). Obesity is also highly prevalent among women with polycystic ovary syndrome (PCOS), affecting 40–80% of those diagnosed (Sam 2007; Vazquez-Martinez et al. 2019). Both PCOS and obesity are associated with elevated leptin, insulin, and testosterone levels, as well as decreased sex hormone-binding globulin.

DNA methylation has been studied in PCOS for over a decade and is known to alter gene expression in blood, ovarian, adipose, and skeletal muscle tissues of affected individuals (Vazquez-Martinez et al. 2019). Increased global DNA methylation has been observed in granulosa cells of patients with PCOS (Xu et al. 2016), alongside differential gene expression patterns in these cells (Kaur et al. 2012; Lan et al. 2015; Coskun et al. 2013). In PCOS rat models, expression of DNMT3A and DNMT3B, along with global DNA methylation, was elevated, while DNMT1 expression remained unchanged (Fig. 2) (Cui et al. 2018).

Metformin, an insulin-sensitizing agent, has shown therapeutic efficacy in PCOS. In rat granulosa cells, PCOS was associated with increased DNMT1 expression and *H19* hypermethylation, leading to *H19* downregulation. Treatment with metformin or sitagliptin normalized DNMT1 and *H19* levels (Wang et al. 2019). DNA methylation of PCOS-related genes, including *Plcg1*, *Camk2b*, *Esr2*, *Ar*, and *Pgr*, was significantly increased in PCOS animal models (Cui et al. 2018) (Fig. 2). One study reported 595 hypomethylated and 323 hypermethylated genes in the umbilical cord blood of neonates born to PCOS mothers, implicating pathways involved in estrogen signaling, mitochondrial function (*APP*, *PARK2*), glucose metabolism (*INS*), GPCR signaling, and inflammation (Lambertini et al. 2017).

Together, these findings indicate that obesity- and PCOS-associated epigenetic alterations converge on dysregulation of genes required for oocyte maturation and ovarian homeostasis. Identification of specific gene targets affected by aberrant DNA methylation should be a priority for future studies. These findings underscore the potential of epigenetic markers as therapeutic targets or biomarkers for obesity- and PCOS-related infertility.

Across female reproductive contexts, obesity is frequently associated with increased DNMT expression and global or locus-specific DNA hypermethylation; however, the magnitude and direction of specific changes vary according to tissue type, disease context, and experimental design. This variability suggests that obesity does not impose a uniform epigenetic response but instead modulates DNA methylation in a cell- and context-dependent manner. Importantly, even when DNMTs are upregulated, methylation output may be constrained by upstream factors such as one-carbon metabolism capacity, oxidative stress, mitochondrial dysfunction, and chromatin accessibility, which together shape the functional impact of DNMT dysregulation. Overall, the available evidence supports a model in which obesity often promotes a hypermethylated epigenetic landscape in female reproductive tissues, driven in part by increased DNMT expression.

### Effects of obesity on DNA methylation and DNA methylation enzymes in oocyte quality and embryo development

Oocyte quality is a critical determinant of fertilization and early embryonic development. Obesity has been consistently associated with impaired oocyte competence, reduced fertilization and implantation rates, and poorer outcomes in assisted reproductive technologies (Bellver et al. 2007; Rittenberg et al. 2011). These defects are partly mediated by obesity-induced alterations in granulosa cell function and changes in follicular fluid composition, including elevated insulin, triglycerides, free fatty acids, proinflammatory cytokines, oxidized LDL, and altered lipid profiles (Bausenwein et al. 2010; La Vignera et al. 2011).

Mechanistic studies indicate that these metabolic perturbations are closely coupled to coordinated changes in DNMT expression and DNA methylation within oocytes. A recent study demonstrated that maternal obesity increases DNMT3A expression and induces global DNA hypermethylation in mouse oocytes, accompanied by reduced expression of the maternal-effect gene *Stella*. Notably, these epigenetic alterations persisted into the F2 generation, providing evidence for a transgenerational epigenetic inheritance mechanism (Chao et al. 2024).

Consistent with these findings, our previous work examined DNMT3B and DNMT3L expression across oocytes, granulosa cells, and ovarian stroma in control and HFD-fed mice (Uysal et al. 2023). Oocytes from HFD-fed mice exhibited increased DNMT3B and reduced DNMT3L expression, whereas granulosa cells showed upregulation of both enzymes; stromal cells displayed reduced DNMT3L levels. These cell-type-specific alterations indicate that obesity disrupts the coordinated epigenetic programming required for proper oocyte maturation and developmental competence.

Obesity-induced changes in DNA methylation have also been observed in oocytes from both HFD-fed and leptin-deficient (ob/ob) mice (Hou et al. 2016). In obese oocytes, methylation of the leptin promoter was increased, whereas methylation of the *Pparα* promoter was reduced. Importantly, oocytes derived from the offspring of mothers with obesity exhibited increased *Pparα* promoter methylation, indicating persistence of obesity-associated epigenetic marks across generations (Ge et al. 2014b; Fig. 2). Pharmacological modulation of metabolic signaling further supports a causal link between metabolism and oocyte epigenetic integrity; treatment with rosiglitazone, an insulin-sensitizing PPAR-γ agonist, improved oocyte developmental competence and partially restored epigenetic balance (Minge et al. 2008).

Beyond the maternal germline, maternal obesity has been associated with increased DNA methylation of the imprinted gene* Peg3 *in the sperm of male offspring, further supporting transgenerational epigenetic transmission (Ge et al. 2014a). Failure to properly establish or maintain germline methylation patterns can result in placental abnormalities, fetal defects, and long-term disease susceptibility (Li et al. 2010).

Among maternal regulators sensitive to metabolic stress, *Stella* has emerged as a critical link between obesity and aberrant oocyte methylation. HFD-induced obesity reduced *Stella* expression, whereas exogenous supplementation rescued methylation defects and restored early embryonic developmental progression following fertilization (Han et al. 2018).

The epigenetic sensitivity of early embryos to maternal metabolic status is further supported by large-animal models. In bovine systems, elevated levels of non-esterified fatty acids (NEFAs), mimicking maternal obesity, during oocyte maturation resulted in increased DNMT3A messenger RNA (mRNA) expression in blastocysts (Van Hoeck et al. 2011). These findings indicate that lipid overload during oocyte maturation can directly influence DNMT regulation and embryonic epigenetic programming.

Increasing evidence suggests that obesity-associated epigenetic alterations are tightly linked to oxidative stress and mitochondrial dysfunction. Mitochondria play a central role in oocyte maturation by regulating bioenergetics, redox homeostasis, and the availability of metabolic intermediates required for one-carbon metabolism, which is essential for DNA methylation reactions (Van Blerkom 2011; Sinclair et al. 2007). In obese conditions, oocyte mitochondrial dysfunction is characterized by reduced membrane potential, impaired ATP production, increased reactive oxygen species generation, and altered mitochondrial DNA content, all of which are associated with diminished oocyte quality and reduced embryo developmental potential (Babayev and Seli 2015).

**Fig. 3:**
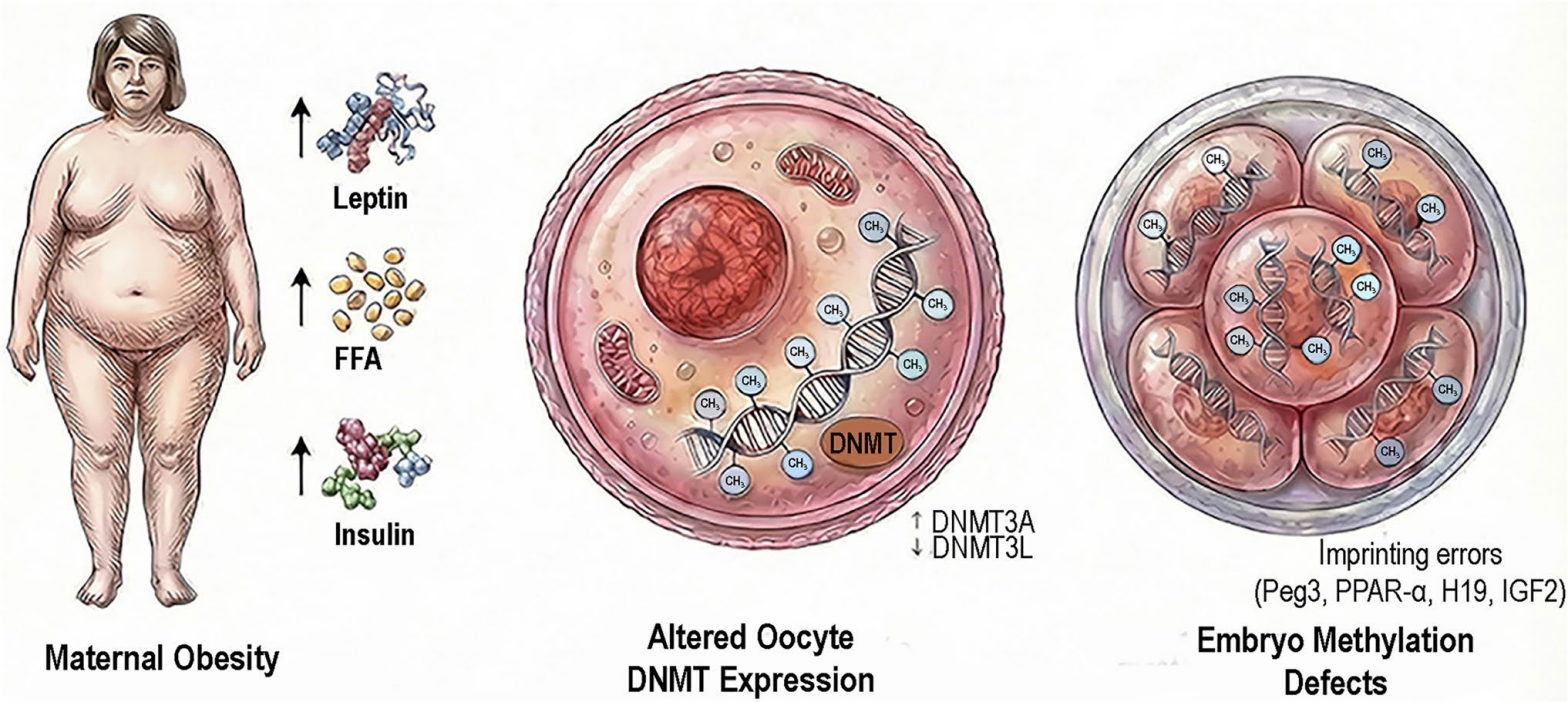
Schematic representation of the effects of maternal obesity on oocyte DNMT expression and early embryonic DNA methylation

Such mitochondrial-driven metabolic perturbations may interfere with methyl donor availability, chromatin organization, and DNMT activity, thereby compromising genomic methylation fidelity during critical windows of oocyte maturation and early embryogenesis (Babayev and Seli 2015; Sinclair et al. 2007; Van Blerkom 2011). The proposed pathway linking maternal obesity to altered oocyte DNMT expression and subsequent embryo DNA methylation defects is schematically illustrated in Fig. 3.

Importantly, when integrated with tissue-level observations, these findings indicate that obesity-associated epigenetic disruption is propagated along the female germline continuum, from ovarian somatic cells to oocytes and early embryos. Functionally, these methylation changes converge on pathways regulating developmental competence, genomic imprinting, and metabolic adaptation, providing a mechanistic bridge between maternal metabolic status and impaired reproductive outcomes.

### Effects of maternal obesity on DNA methylation and DNA methylation enzymes during pregnancy

Fetal ovarian development is highly sensitive to the intrauterine environment, and maternal obesity together with gestational diabetes (GD) can induce persistent epigenetic alterations that influence both reproductive and metabolic trajectories later in life (Sinha et al. 2019). Offspring of dams exposed to GD exhibit an increased risk of obesity, metabolic syndrome, and cardiovascular disease, highlighting the long-term developmental consequences of maternal metabolic dysregulation.

In a mouse model, expression of cocaine- and amphetamine-regulated transcript (*CART*) was significantly elevated in the ovaries of offspring derived from GD pregnancies, an effect attributed to global DNA hypomethylation. This dysregulation was associated with abnormal folliculogenesis and reduced fertility (Sinha et al. 2019; Fig. 2). These findings indicate that disrupted intrauterine glucose homeostasis can directly reprogram ovarian epigenetic profiles in the developing fetus.

Complementary evidence from human studies further supports obesity-associated epigenetic remodeling at the maternal–fetal interface. Genome-wide analyses indicate that maternal obesity and gestational diabetes can induce persistent methylome reprogramming in offspring, extending beyond birth and influencing metabolic and developmental pathways (Alba-Linares et al. 2023). These findings reinforce the concept that maternal metabolic disturbances exert long-lasting epigenetic effects during critical windows of fetal development.

In placental tissues obtained from women with and without obesity, hypomethylation of the adiponectin promoter and hypermethylation of its receptor were observed on the maternal side, whereas the fetal side exhibited increased methylation of the leptin promoter (Nogues et al. 2019). Such asymmetric methylation patterns suggest compartment-specific epigenetic responses that may disrupt placental endocrine signaling and nutrient sensing.

**Fig. 4:**
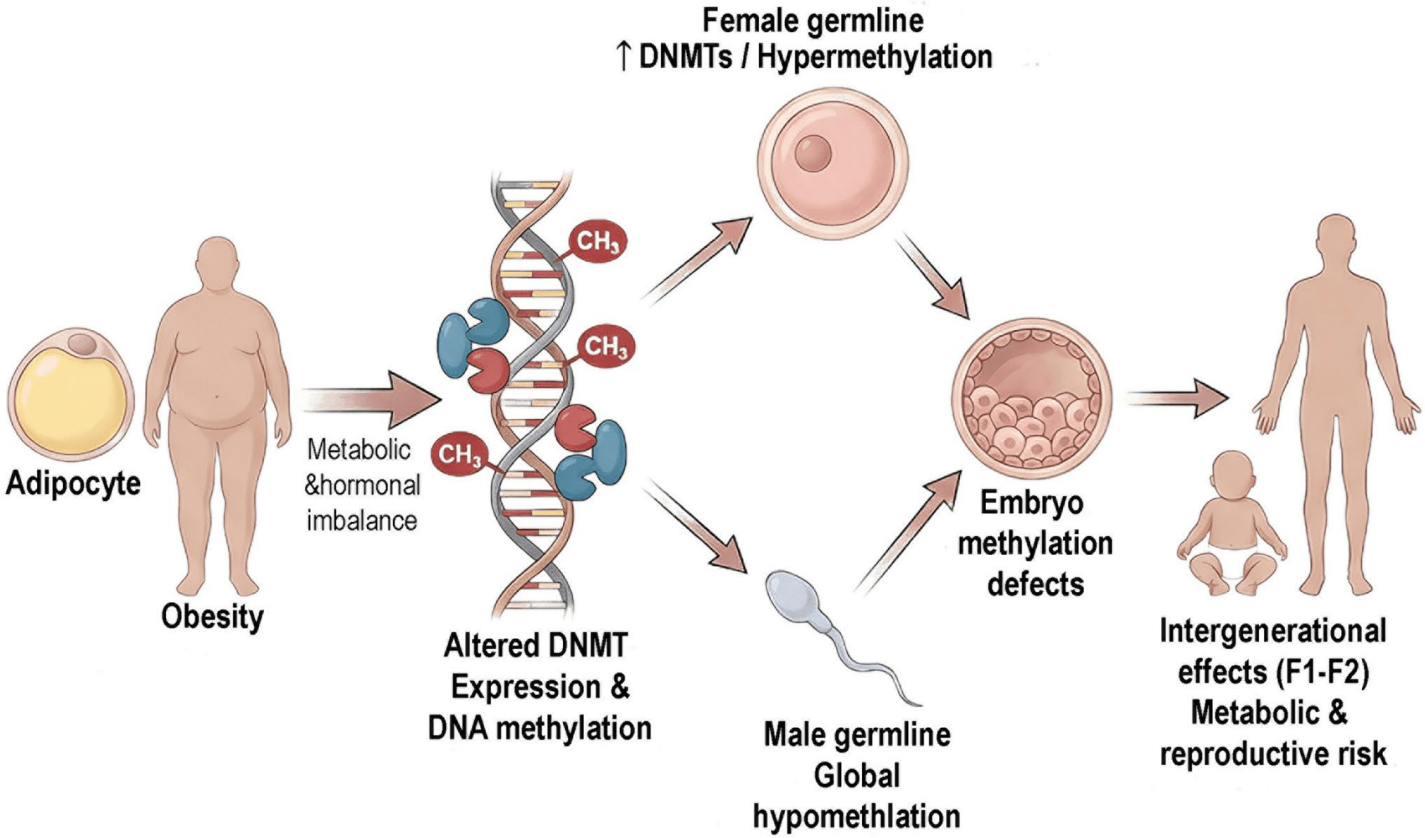
Summary model of obesity-induced DNMT dysregulation and epigenetic outcomes in reproduction. Obesity alters DNA methyltransferase (DNMT) expression and global DNA methylation in female and male germ cells in a sex-specific manner. In female individuals, obesity is predominantly associated with DNMT upregulation and global DNA hypermethylation in oocytes, whereas in male individuals, it is mainly characterized by DNA hypomethylation in the testis and sperm. These epigenetic alterations may disrupt gamete quality and early embryonic methylation patterns, and contribute to transgenerational metabolic and reproductive effects

Collectively, these data demonstrate that maternal obesity induces site-specific and compartment-specific DNA methylation changes not only in fetal tissues but also in pregnancy-associated structures critical for developmental programming (Fig. 4). Importantly, these observations extend obesity-associated DNMT dysregulation and DNA methylation remodeling beyond adult gonadal tissues to include prenatal windows of susceptibility. In this context, pregnancy emerges as an additional window in which maternal metabolic imbalance can reshape epigenetic programming with potential long-term consequences for offspring health.

Notably, the hypermethylation-dominated patterns observed across female reproductive and pregnancy-associated contexts contrast with the predominantly hypomethylated profiles reported in the male reproductive system. This divergence underscores a sexually dimorphic epigenetic response to metabolic stress, which is explored further in the following section.

## The effect of obesity on the male reproductive system

In contrast to the female reproductive system, obesity in male individuals is associated with distinct epigenetic remodeling characterized predominantly by global and locus-specific DNA hypomethylation. This pattern reflects a sexually dimorphic epigenetic response to metabolic stress rather than a uniform shift in DNA methylation across reproductive tissues.

Male obesity is closely associated with hypogonadism. Most men with obesity exhibit a characteristic hormonal profile marked by elevated estrogen and leptin levels together with reduced testosterone, follicle-stimulating hormone (FSH), sex hormone-binding globulin (SHBG), ghrelin, and inhibin B. This endocrine imbalance is largely driven by increased aromatase activity in expanded white adipose tissue, which enhances the conversion of androgens to estrogens and induces negative feedback at the hypothalamic–pituitary level, thereby suppressing gonadotropin secretion.

**Fig. 5:**
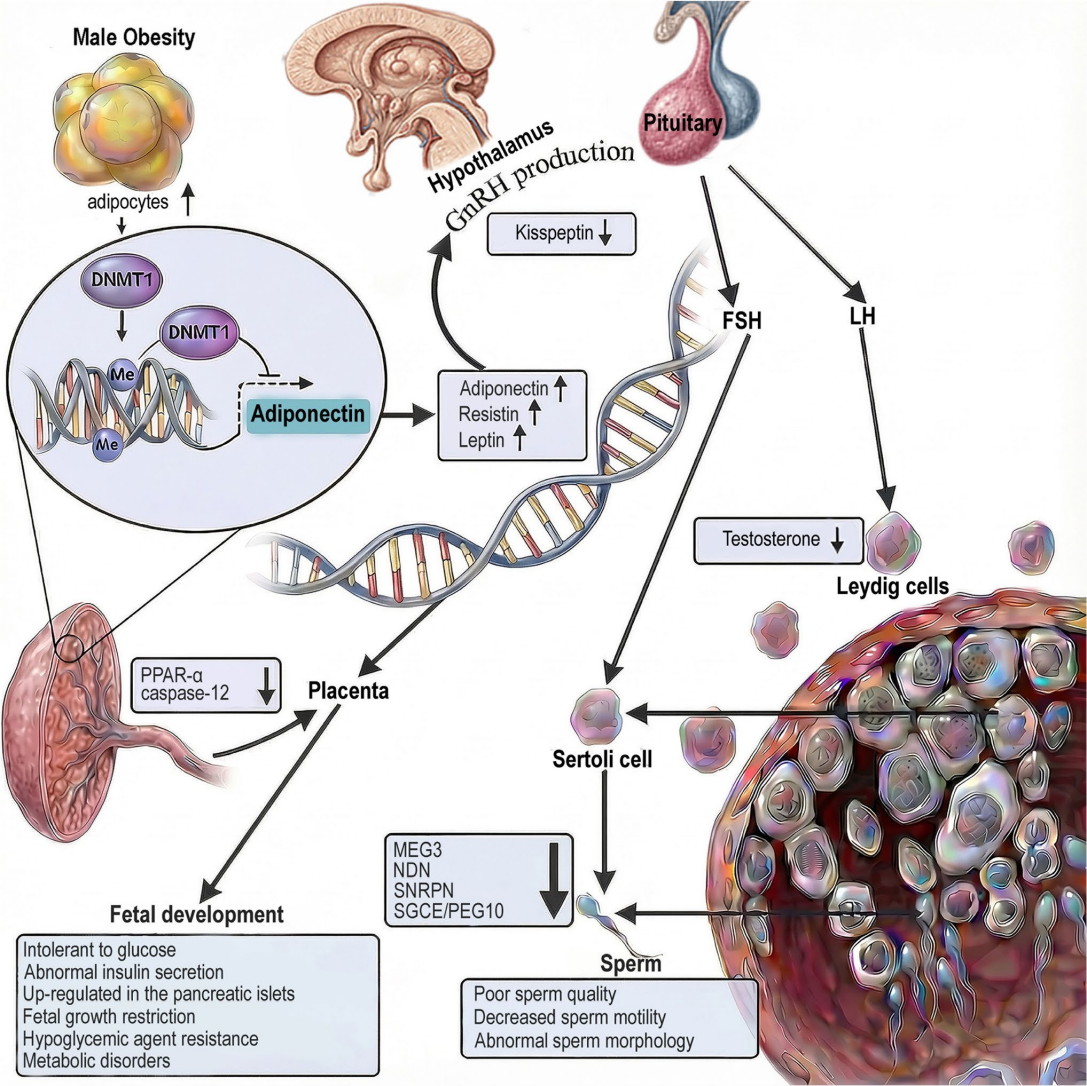
Increased adipokines and reduced kisspeptin levels impair the HPG axis and spermatogenesis. Hypomethylation of imprinted genes in sperm and placental alterations may impact offspring health. ↑, increase; ↓, decrease. *HPG* hypothalamic–pituitary–gonadal, *LH* luteinizing hormone, *FSH* follicle-stimulating hormone, *GnRH* gonadotropin-releasing hormone, *MEG3* maternally expressed gene 3, *NDN* necdin, *SNRPN* small nuclear ribonucleoprotein polypeptide N, *SGCE* sarcoglycan epsilon, *PEG10* paternally expressed gene 10, *Casp12* caspase-12, *PPAR-α* peroxisome proliferator-activated receptor alpha

Consequently, the production of gonadotropin-releasing hormone (GnRH) and luteinizing hormone (LH) is reduced, further impairing testosterone synthesis and disrupting hypothalamic–pituitary–gonadal (HPG) axis homeostasis (Liu and Ding 2017). Beyond hormonal dysregulation, accumulating evidence indicates that obesity-associated metabolic stress also reshapes DNA methylation dynamics within developing germ cells. The hormonal and epigenetic pathways linking male obesity to impaired spermatogenesis are summarized in Fig. 5.

Reduced circulating levels of kisspeptin, an essential neuropeptide that stimulates GnRH secretion, have been reported in men with obesity. This reduction has been proposed to arise, at least in part, from DNA methylation-mediated transcriptional repression, identifying kisspeptin signaling as a potential epigenetic target of obesity-related metabolic disturbance (Davidson et al. 2015). Decreased GnRH secretion results in reduced LH and FSH release, impairing Leydig and Sertoli cell function and contributing to decreased testosterone production. Under these conditions, estrogen levels may exceed testosterone levels (Hohos and Skaznik-Wikiel 2017).

In parallel, hormonal imbalances, chronic inflammation, and oxidative stress induced by obesity adversely affect the testes and epididymis, organs essential for spermatogenesis and sperm maturation. These interconnected disturbances converge to impair sperm concentration, motility, and morphology, thereby contributing to male subfertility (Liu and Ding 2017; Davidson et al. 2015; Fullston et al. 2013). Importantly, these physiological impairments are increasingly interpreted within an epigenetic framework linking metabolic stress to germline methylation instability.

Epidemiological studies further indicate that elevated paternal body mass index (BMI) is associated with prolonged gestation (Nguyen et al. 2007; Ramlau-Hansen et al. 2007), reduced clinical pregnancy rates (Bakos et al. 2011; Merhi et al. 2013), and increased metabolic risk in offspring (Fullston et al. 2015; Potabattula et al. 2019).

Importantly, emerging evidence indicates that sperm epigenetic profiles are dynamic and responsive to metabolic interventions. Following bariatric surgery, substantial remodeling of sperm DNA methylation has been reported, particularly at loci associated with metabolic regulation, appetite control, and central nervous system function (Donkin et al. 2016). These alterations were detectable shortly after weight loss and persisted up to 1-year post-surgery, underscoring the sensitivity of the sperm epigenome to metabolic state.

Collectively, and in clear contrast to the predominantly hypermethylated patterns observed in female reproductive tissues, obesity in male individuals is more frequently associated with global and locus-specific DNA hypomethylation (Fig. 4). This divergence reinforces the central premise of this review that DNMT dysregulation and altered DNA methylation constitute mechanistic mediators of obesity-associated reproductive dysfunction.

### Effects of obesity on DNA methylation and DNA methylation enzymes in the testis

DNA methylation is indispensable for normal spermatogenesis, particularly for the establishment of paternal genomic imprints and X-chromosome regulation. This epigenetic programming begins in primordial germ cells and continues through spermatogonia to mature spermatozoa (Goto and Monk 1998; Ooi and Henikoff 2007; Boissonnas et al. 2013). Throughout this process, de novo and maintenance methylation events must be precisely coordinated to preserve germ cell identity, genome stability, and reproductive competence. Disruption of DNA methylation homeostasis is therefore expected to exert profound effects on germ cell development and epigenetic integrity.

Consistent with this framework, HFD-induced obesity has been shown to alter DNA methylation patterns in testicular tissue, with some of these modifications being transmitted to offspring (de Castro Barbosa et al. 2016). These findings indicate that obesity-associated metabolic stress can interfere with the epigenetic reprogramming events that are fundamental to male germline development.

In our previous studies, we demonstrated that HFD-induced obesity significantly alters both DNMT expression and global DNA methylation during testicular spermatogenesis in a mouse model. DNMT1 and DNMT3A levels were significantly elevated, whereas global DNA methylation was unexpectedly decreased in the testes of HFD-fed mice (Sukur et al. 2023). These observations align with those of Fullston et al., who similarly reported reduced global DNA methylation in total testicular tissue following HFD exposure (Fullston et al. 2013).

However, not all studies report identical molecular responses. Deshpande et al. observed decreased DNMT1 mRNA levels in HFD-fed rats, while DNMT3A, DNMT3B, and DNMT3L expression remained unchanged (Deshpande et al. 2021). Such discrepancies likely reflect differences in species, diet composition, duration of metabolic stress, and the specific germ cell populations analyzed.

Independent of obesity models, genetic evidence underscores the essential role of DNMT-mediated methylation in male fertility. DNMT3L knockout mice exhibit reduced testis size and litter numbers (Vlachogiannis et al. 2015). Although DNMT3L-deficient animals are viable, both males and females display infertility due to impaired germ cell development (Bourc’his et al. 2001; Hata et al. 2002). These findings reinforce the concept that perturbation of DNMT-regulated methylation pathways, whether genetically or metabolically induced, can severely compromise reproductive function.

Taken together, available evidence indicates that, unlike the predominantly hypermethylated patterns observed in female reproductive tissues, testes and developing male germ cells more frequently exhibit global or locus-specific DNA hypomethylation in the context of obesity. Notably, several studies describe an imperfect correspondence between DNMT abundance and global DNA methylation levels in obese testes. This pattern suggests that increased DNMT expression does not necessarily translate into proportional methylation output. Rather, the functional consequences of DNMT dysregulation appear to be shaped by upstream regulatory constraints, including methyl donor availability, oxidative stress, chromatin accessibility, and the temporal dynamics of epigenetic reprogramming.

Because these testicular alterations ultimately influence the sperm epigenome, the following subsection focuses specifically on obesity-associated DNA methylation and DNMT dynamics in mature sperm.

### Effects of obesity on DNA methylation and DNA methylation enzymes on sperm

Spermatogenesis is a highly coordinated developmental process characterized by extensive epigenetic reprogramming, including dynamic remodeling of DNA methylation landscapes. Disruption of these methylation dynamics can compromise germ cell differentiation, chromatin organization, and sperm function. Consistent with this vulnerability, HFD-induced obesity in mice has been shown to significantly reduce global DNA methylation in both testicular tissue and developing spermatids (Fullston et al. 2013) (Fig. 1).

Beyond DNA methylation alone, accumulating evidence indicates that the sperm epigenome is inherently multilayered, comprising DNA methylation, retained histones, and diverse populations of regulatory RNAs. These interconnected epigenetic components provide complementary routes through which paternal metabolic, inflammatory, and oxidative stressors may influence fertilization competence and early embryonic programming (Lee and Conine 2022). In particular, sperm chromatin features, including histone retention and associated modifications, have been proposed to modulate gene regulation during the preimplantation period (Dodd and Luense 2024).

Recent syntheses further suggest that selected paternal epigenetic signatures can persist after fertilization in functionally meaningful forms, contributing to early embryonic transcriptional regulation (Lismer and Kimmins 2023). In parallel, sperm-borne small noncoding RNAs have emerged as environmentally responsive mediators capable of transmitting information about paternal metabolic status (Liu and Sharma 2023). Together, these findings support a model in which obesity-associated alterations are not restricted to sperm DNA methylation but involve coordinated remodeling across multiple epigenetic layers.

Human studies provide direct evidence that obesity is associated with altered sperm DNA methylation. In a cohort study involving 23 men with overweight/obesity and 44 normal-weight controls, sperm from the overweight/obese group exhibited reduced methylation at several imprinted genes, including *MEG3*, *NDN*, *SNRPN*, and *SGCE/PEG10* (Soubry et al. 2016) (Fig. 1). Similarly, genome-wide analyses have identified significant methylation differences at more than 3000 CpG sites in sperm from men with obesity, with enrichment in genes associated with stem cell regulation, transcriptional control, and oncogenic pathways (Keyhan et al. 2021).

Consistent with these observations, recent reviews increasingly position sperm DNA methylation changes, together with other epigenetic layers, as potentially informative biomarkers and mechanistic mediators of male-factor infertility, while emphasizing substantial heterogeneity across cohorts and analytical platforms (Caroppo and Skinner 2024; Hosseini et al. 2024; Crafa et al. 2024). Lifestyle-focused syntheses further identify paternal BMI and obesity among modifiable exposures capable of reshaping sperm epigenetic signatures with implications for embryo development and offspring health (Akhatova et al. 2025).

Mechanistically, oxidative stress has emerged as a key mediator linking obesity to sperm epigenetic instability. Obesity-associated oxidative stress is closely coupled to mitochondrial dysfunction, which contributes to impaired sperm motility, DNA damage, and reduced fertilization capacity (Jing et al. 2023). Contemporary evidence indicates that oxidative stress-associated pathways can perturb DNA methylation, histone modifications, and small RNA profiles during spermatogenesis (Sudhakaran et al. 2024; Kaltsas et al. 2025). Broader paternal metabolic exposures, including diet quality and obesity, are therefore increasingly discussed as preconception determinants of sperm epigenetic programming with potential downstream effects on placental function and offspring metabolic phenotypes (Tahiri et al. 2024; Skerrett-Byrne et al. 2025).

At the cellular level, obesity-associated alterations in DNMT expression during spermatogenesis provide mechanistic context for these sperm methylation changes. In our previous study, DNMT1 expression in spermatogonia was comparable between groups; however, significant increases were observed in spermatocytes and round spermatids of HFD-fed mice. Although DNMT3A followed a similar trend, global DNA methylation levels were significantly reduced in round and elongated spermatids, indicating a disconnect between DNMT abundance and methylation output (Sukur et al. 2023). Notably, DNMT1 and DNMT3A proteins were absent in elongated spermatids despite elevated global methylation, supporting the interpretation that DNMT activity primarily operates during earlier germ cell stages. These findings are consistent with those of Fullston et al. (2013).

Supporting this stage-specific model, DNMT localization and expression patterns differ across germ cell populations. DNMT1 has been detected in both the nucleus and cytoplasm of spermatogonia and round spermatids, whereas its absence has been associated with spermatogenic arrest (Omisanjo et al. 2007). DNMT3B mRNA is not expressed in elongated spermatids in humans (Marques et al. 2011), and similar stage-restricted expression has been described in mice (La Salle and Trasler 2006). Additionally, inactivation of DNMT3L results in impaired spermatogenesis (Hata et al. 2006), underscoring the importance of DNMT-mediated methylation during earlier developmental windows.

Further evidence for obesity-associated epigenetic remodeling in sperm derives from imprinting control regions. Methylation at the *MEG3-IG* and *H19* differentially methylated regions (DMRs) has been reported to be modestly altered in sperm from men who are overweight (Soubry et al. 2016). Potabattula et al. identified sex-specific correlations between paternal BMI and DNA methylation patterns at *MEG3-IG DMR*, *HIF3A*, and *IGF2 DMR0* in offspring, suggesting that paternal obesity may influence sperm DNA methylation and contribute to epigenetic reprogramming in the next generation (Potabattula et al. 2019).

Mechanistic support for sperm-borne epigenetic signaling has expanded further with evidence that specific sperm RNAs can be diet-induced and transmitted during fertilization, providing a complementary route to DNA methylation-based inheritance models (Tomar et al. 2024). Consistent with this interpretation, foundational and contemporary reviews of obesity-related epigenetic inheritance summarize potential mechanisms through which germline epigenetic remodeling may transmit altered metabolic susceptibility across generations (King and Skinner 2020).

Integration of human and animal studies indicates that obesity-associated remodeling of the sperm epigenome frequently involves hypomethylation at imprinted and metabolically relevant loci. These alterations provide a mechanistic basis linking paternal metabolic status to changes in fertilization competence and early embryonic programming. When contrasted with female reproductive tissues, where obesity is more commonly associated with DNMT upregulation and DNA hypermethylation, sperm-centered observations highlight a pronounced sex-specific divergence in DNA methylation responses.

This divergence underscores the importance of considering germ cell developmental context, chromatin organization, and metabolic regulation when interpreting obesity-induced epigenetic outcomes. These sperm-associated epigenetic signatures may, in turn, contribute to altered embryo development and offspring health, as discussed in the following section.

### Effects of paternal obesity on DNA methylation and DNA methylation enzymes in embryo development

Experimental and epidemiological evidence indicates that paternal obesity can induce DNA methylation alterations in sperm that are subsequently propagated to embryonic and somatic tissues of the offspring. These findings support the concept that paternal metabolic status contributes directly to early developmental programming through epigenetic mechanisms.

For example, Ng et al. demonstrated that female offspring (F1) of HFD-fed male rats developed glucose intolerance and abnormal insulin secretion. These metabolic disturbances were associated with hypomethylation and increased expression of *Il13ra2* in pancreatic islets (Ng et al. 2010) (Fig. 1), providing early functional evidence that paternal diet-induced obesity can reshape offspring metabolic phenotypes via epigenetic remodeling.

Consistent with these observations, paternal obesity in C57BL/6 mice has been linked to fetal growth restriction (Baker et al. 2015; Kohl et al. 2012), a developmental condition associated with increased susceptibility to obesity and metabolic disease later in life. Placental tissues derived from offspring of fathers with obesity exhibited reduced expression of *Pparα* and *Casp12* (Binder et al. 2015), suggesting that paternal metabolic disturbances may influence placental gene regulation, potentially through altered sperm-borne epigenetic signals.

Beyond germline-mediated effects, obesity-associated DNA methylation alterations have also been detected across multiple somatic tissues involved in metabolic regulation. Increased methylation at CpG site 437 of the *PPARγ2* promoter, for instance, has been reported in omental tissue of individuals with obesity (Barres and Zierath 2011) (Fig. 5). Although these findings primarily reflect systemic metabolic dysregulation, they reinforce the broader principle that obesity is accompanied by pervasive epigenetic remodeling across tissues.

Several studies further indicate that paternal obesity preferentially affects the methylation of imprinted and metabolically relevant genes. Obesity-induced alterations in DNA methylation at imprinted loci, including *Peg3*, *Peg9*, and *Peg10*, as well as the nutrient transporter gene *Slc38a2*, have been observed in F1 placentas (Mitchell et al. 2017). Aberrant methylation of the *Peg9* promoter was also reported, suggesting instability at imprinting control regions. Similarly, the *Nr1h3* locus, encoding a nuclear receptor involved in lipid metabolism, was identified as responsive to HFD exposure in germ cells and later found to be altered in the liver of F2 females (Park et al. 2018), supporting the possibility of transgenerational inheritance.

In human cohorts, differential DNA methylation patterns have been documented in children born to parents with obesity (Soubry et al. 2015), further substantiating the translational relevance of paternal obesity-associated epigenetic effects.

Collectively, these observations support a model in which paternal obesity modifies sperm epigenetic programming, leading to altered embryonic and placental DNA methylation landscapes that shape offspring metabolic and developmental trajectories. Importantly, these effects are increasingly interpreted within a framework of intergenerational and potentially transgenerational epigenetic inheritance.

Environmental exposures such as diet and obesity are therefore recognized as capable of inducing heritable changes in DNA methylation without altering DNA sequence (Abdul et al. 2017). Contrary to earlier assumptions that epigenetic marks are largely erased between generations, accumulating evidence suggests that selected modifications may persist and contribute to disease susceptibility across multiple generations (Sharma 2017; Painter et al. 2008).

**Fig. 6:**
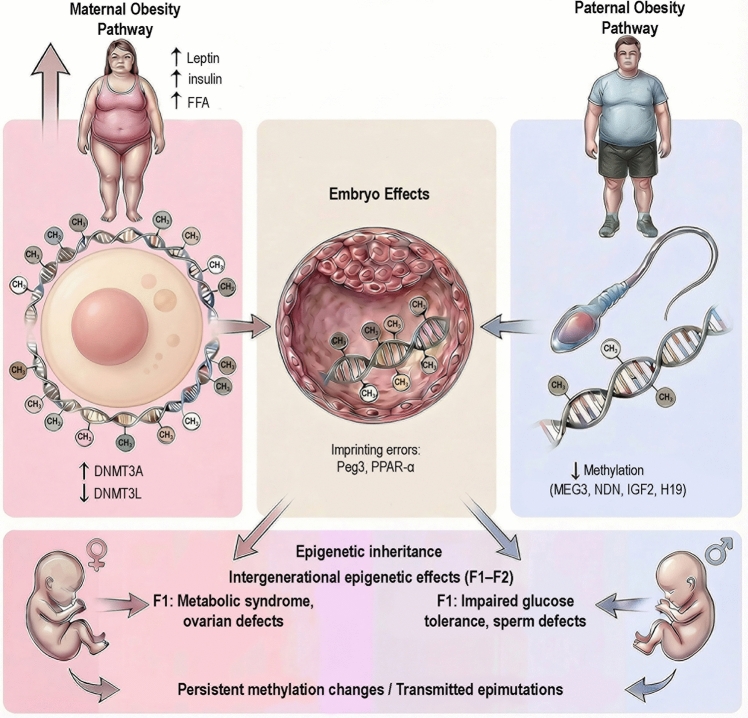
Summary model illustrating the differential effects of maternal and paternal obesity on DNMT regulation, germline DNA methylation, and intergenerational epigenetic outcomes. Maternal obesity is associated with altered oocyte DNMT expression and hypermethylation, whereas paternal obesity is predominantly linked to sperm DNA hypomethylation. These sex-specific epigenetic alterations contribute to embryonic imprinting defects and metabolic and reproductive outcomes in subsequent generations (F1–F2)

The combined maternal and paternal contributions to embryo epigenetic programming and intergenerational inheritance are summarized in Fig. 6, which outlines proposed routes through which altered gametic DNA methylation and sperm-borne epigenetic information influence early embryonic development.

## Conclusions

The evidence synthesized in this review positions DNMT-mediated DNA methylation as a central molecular interface linking obesity-associated metabolic and endocrine disturbances to reproductive dysfunction. Across female reproductive tissues, including the ovaries, oocytes, placenta, and pregnancy-associated contexts, obesity is frequently associated with increased DNMT expression and a shift toward a hypermethylated epigenetic landscape. These alterations converge on pathways governing folliculogenesis, oocyte competence, implantation, and early embryonic development, providing a mechanistic framework for the reduced fertility observed in female individuals with obesity.

In contrast, studies of the male reproductive system more frequently describe global and locus-specific DNA hypomethylation in the testes, developing germ cells, and sperm, despite variable or even elevated DNMT expression. This divergence underscores a sexually dimorphic epigenetic response to metabolic stress and suggests that intrinsic differences in chromatin organization, germ cell developmental dynamics, and metabolic sensitivity shape how obesity is translated into DNA methylation outcomes. Across sexes, an emerging theme is the imperfect correspondence between DNMT abundance and global methylation output. This observation indicates that upstream regulatory constraints, including altered one-carbon metabolism, oxidative stress, mitochondrial dysfunction, and cell-type-specific regulatory mechanisms, critically modulate the functional consequences of DNMT dysregulation.

Beyond direct effects on gametes, obesity-associated epigenetic alterations may be propagated to early embryos and, in certain contexts, across generations. These findings extend the implications of DNA methylation remodeling from individual reproductive capacity to offspring metabolic and reproductive health, positioning germline epigenetic programming as a potential mediator of intergenerational disease susceptibility.

Despite substantial progress, several important knowledge gaps remain. These include clarifying causal relationships between specific methylation changes and defined reproductive phenotypes, determining the reversibility of obesity-induced epigenetic alterations, disentangling the relative contributions of DNA methylation and other epigenetic regulatory layers, and establishing standardized experimental frameworks across dietary models and analytical platforms. Greater integration of longitudinal, mechanistic, and intervention-based studies will be particularly important. Addressing these challenges through single-cell, spatial, and multiomic approaches will be essential for refining mechanistic understanding.

Taken together, the integrated evidence underscores DNMT dysregulation and DNA methylation remodeling as fundamental mechanisms linking obesity to reproductive dysfunction. This conceptual framework advances current understanding of obesity-associated infertility and highlights epigenetic features as promising candidates for biomarker development and therapeutic intervention.

## Data Availability

No datasets were generated or analyzed during the current study.
