# Correction: Effects of obesity on DNA methylation and DNA methyltransferases in the reproductive system

**DOI:** 10.1007/s00418-026-02493-7

**Published:** 2026-06-13

**Authors:** Gozde Sukur, Ozgur Cinar, Fatma Uysal Cinar

**Affiliations:** 1 Department of Molecular Biology and Genetics, Faculty of Science, Gebze Technical University, 41400 Gebze, Kocaeli Turkey; 2 Department of Histology and Embryology, Ankara University School of Medicine, 06080 Ankara, Altindag Turkey; 3 Department of Histology and Embryology, Ankara Medipol University School of Medicine, 06080 Ankara, Altindag Turkey

**Correction: Histochemistry and Cell Biology (2026) 164:30**

10.1007/s00418-026-02481-x

In this article, the following author names Gozde Sukur, Ozgur Cinar, Fatma Uysal Cinar were incorrectly written as Gözde Şükür, Özgür Çınar, Fatma Uysal Çınar.

The original article has been corrected.

